# Some Queer Definitions in Hospital Diet

**Published:** 1911-09-30

**Authors:** Thos. Alex. Cook

**Affiliations:** Chairman of General Committee of Management. West Ham Hospital.


### SOME QUEER DEFINITIONS IN HOSPITAL DIET.

*To the Editor of* The Hospital.

September 25.

*To the Editor of* The Hospital.

Sir,—Much interest has been aroused by the correspondence in your influential columns with regard to the gratuitous supply of tea, sugar, and butter to in-patients in hospitals. I think I should rather have said, butter, tea, and sugar to put them in order of costliness.

It seems to me that with regard to sugar and butter they are diet-essentials. With regard to tea, much difference of opinion exists, and it is not for a layman to enter into a controversy which is eminently one for specialists to argue. But as it seems generally to be admitted that the contented mind is an assistance, if not a necessity, to good, bodily recovery, then the "cup that cheers" might perhaps be given a place as a clinical aid.

At the West Ham and Eastern General Hospital we decided some years ago not only that treatment and diet should be absolutely free to deserving cases, but we went a step further. We used to allow friends to bring presents of fresh eggs, butter, and tea; this permission we absolutely withdrew for various reasons. We found that visitors, under cover of the parcels of eggs or tea, frequently smuggled undesirable diet or beverages. We found that jealousies and petty feelings were aroused. One knows that in any ward there may always be one at least cantankerous soul, and such a one is apt to accuse the nurse or sister of giving his new laid egg to another patient, or supplying him with hospital tea instead of his own. The sisters' duties and worries are increased. The food provided by the patients and their friends may not be suitable, but grievances are aroused if it is withheld.

All the above are cogent reasons on the domestic side. But from the administrative side, as a matter of principle, is it wise to allow patients or their friends to provide anything required during residence in the wards of a charitable institution? Nearly every hospital now has an almoner or officer to see that the charity is not abused. Surely if only those are admitted who cannot afford treatment, it follows that to force them to provide diet is an added hardship during enforced idleness. If it is the father, he may know that his family are living on a Club allowance, perhaps less than half of his usual weekly earnings; if a mother, she is concerned that paid help may have to be employed on washing day; and such considerations would make the tax of providing tea, butter, and eggs seem an additional hardship.

It may be said: "Yes; but take accident cases. A man or woman well able to pay for treatment may meet with so serious an accident as to have to be conveyed to the nearest hospital. Surely he or she could provide themselves with tea, butter, and eggs." I am happy to say that in the great majority of such cases our experience is that the grateful patient or the friends give a donation in accordance with their means. I am informed that tea costs the hospital about 4d. per week per in-patient; sugar from 1d. to 1½d.; butter 6d. for the in-patient on full diet. These are estimates, but, I hope, near ones. Thus, if a patient were in for four weeks, and his tea, sugar, and butter (bought retail) cost him, say, 2s. a week, he might withhold a donation of a guinea, and the hospital would be worse off.

I am afraid I am straining the hospitality of your columns, but one last thought. If necessity is the key to the patients' admission to hospital, should not uniformity be the keynote of their entertainment therein?

Yours faithfully,
Thos. Alex. Cook,
Chairman of General Committee of Management.

West Ham Hospital.
September 25.

